# Sociodemographic Characteristics and Risk Factors for Childhood Poisoning Reported by Parents at a Tertiary Care Teaching Hospital

**DOI:** 10.7759/cureus.13313

**Published:** 2021-02-12

**Authors:** Ali A Alhaboob

**Affiliations:** 1 Pediatrics, King Saud University, Riyadh, SAU

**Keywords:** childhood injury, childhood poisoning, household chemicals, ingestion of toxic materials, cleaning materials, petrochemical materials, acute poisoning, drug intoxication, hydrocarbon ingestion, poisonous materials

## Abstract

Background

Childhood poisoning is a major health problem. Mostly, it is accidental and associated with low morbidity and mortality. The association between sociodemographic factors and childhood injury rates could be used for improvement to prevent and reduce such injuries. Childhood poisoning is preventable through appropriate education and judicious storage of drugs and household chemicals that might help in reducing and eliminating the accidental ingestion of toxic materials at home.

Objectives

To recognize the potential risk factors that might be associated with childhood home poisoning in Riyadh City, Kingdom of Saudi Arabia.

Design

A survey-based questionnaire study.

Setting

A tertiary care teaching hospital in Riyadh City.

Patients and methods

A structured questionnaire was created, which included questions on the poisoning incidence, home medication history, and possible risk factors for poisoning and the sociodemographic characteristics, and was disseminated to individuals who visited the King Khalid University Hospital.

Main outcome measures

Demographic characteristics of participants and risk factors related to childhood poisoning.

Results

The study included 152 randomly selected participants, 62 men (40.79%) and 90 women (59.21%). Self-ingestion was reported to be the most common mode of poisoning 28/44 (63.6%). The appearance of clinical manifestations suggesting poisoning was reported to be the most frequent method of discovery of children poisoning 20/44 (45.5%). Thirty-six out of the 44 respondents (81.8%) with a positive history of childhood poisoning in their family transferred their children to a hospital immediately. Drugs were the most common causative agent reported for poisoning among the respondents 21/44 (47.7%).

Conclusion

Accidental and non-intentional self-ingestion still presents as a major mode of childhood home poisoning. Despite the significant advancement in the lifestyle among the majority of Saudi Arabian regions, especially the capital city Riyadh, childhood poisoning remains a significant cause of morbidity and possible mortality. Creating health education and prevention programs might help to prevent such serious preventable problems.

Limitations

The limited number of participants may not reflect the whole population living in Riyadh City, hence, interpretation of the study results might be taken cautiously.

Conflict of interest

There was no conflict of interest.

## Introduction

Childhood poisoning still presents as a major health problem worldwide, which is mostly accidental and, fortunately, associated with low morbidity and mortality [1]. It is a significant cause of emergency department visits and hospital admissions [2]. Children aged <6 years are the most commonly affected age group, accounting for up to 60% of all cases of childhood poisoning [3]. The majority of childhood ingestions and poisoning occur at home [4].

The observed association between sociodemographic factors and childhood injury rates could be used for improvement aiming to prevent and/or reduce such injuries in general [5]. Many factors have been considered risk factors for childhood poisoning, including family stress, residential circumstances, number of family members, awareness of prevention methods, and socioeconomic status or child caregiver characteristics such as less proximal parental supervision or psychiatric distress [6]. Moreover, the types of poisoning and causative agents vary greatly among different areas worldwide. Recognizing the pattern and different sociodemographic factors implicated in childhood poisoning at the local and/or national levels might help in the strategic planning for children's healthcare services and protection programs to reduce the morbidities and mortalities from such incidents.

Childhood poisoning is preventable through appropriate education and judicious storage of drugs and household chemicals, which can serve to reduce the incidence of or eliminate accidental ingestion of toxic materials at home [7].

Several studies related to accidental childhood poisoning are readily available from different regions of the Kingdom of Saudi Arabia [8-11]. However, there are currently no available data regarding childhood poisoning pattern and prevention awareness in Riyadh City, Kingdom of Saudi Arabia.

This survey aimed to recognize the potential risk factors that might be associated with higher risks of childhood home poisoning in Riyadh City, Kingdom of Saudi Arabia.

## Materials and methods

This questionnaire study was conducted at King Khalid University Hospital, King Saud University, Riyadh City, Kingdom of Saudi Arabia.

A questionnaire sheet was structured in Arabic language including the following queries: 1) sociodemographic data, including parents’ age, educational status, and work; residential area; home type; number of family members; children caregiver; presence of a housemaid; economic status; marital status; social problems; and availability of a family physician; 2) home medication history, including presence of medications at home, medication type, storage place, secure closure, and accessibility for children; 3) other materials that are potential hazards for childhood poisoning; 4) awareness on preventing or managing childhood poisoning; and 5) incidence and possible risk factors for childhood home poisoning, mode of incidence, mode of discovery, and actions taken.

The study included 152 participants who visited the Pediatric Emergency and Outpatient Departments at King Khalid University Hospital, King Saud University Medical City, Riyadh City, Kingdom of Saudi Arabia for different reasons. The study objectives and confidentiality of the collected data were explained to participants, after which, a consent was obtained from those who agreed to participate.

Statistical analysis

The collected questionnaire data were analyzed using the Statistical Package for the Social Sciences version 12.0 (SPSS Inc., Chicago, USA). Frequencies were calculated for qualitative data and means (Standard Deviations [SDs]) and medians for quantitative data. Paired t-test for quantitative data and Chi-squared test for qualitative data were used to assess the significances in the comparison of the different variables.

## Results

Table 1 shows the sociodemographic characteristics, home medication history, poisoning incidence, and possible risk factors among the respondents.

**Table 1:** Sociodemographic and studied characteristics of respondents * Grandmother or father; ** More than one form of medicine might be present at the same time; *** More than five members

Sociodemographic characteristics	Number	Percentage
Total number of participants	152	
Informant		
Man	62	40.79%
Woman	90	59.21%
Marital status		
Married	134	88.20%
Divorced	4	2.60%
Second wife	14	9.20%
Father’s educational status		
Educated	141	92.20%
Non-educated	11	7.20%
Father’s work		
Not working	7	4.60%
Employee	134	87.60%
Free work	11	7.20%
Caregiver		
Mother	105	69.10%
Others^*^	47	30.90%
Mother’s educational status		
Educated	84	54.60%
Non-educated	68	44.40%
Housemaid		
Yes	69	45.40%
No	83	54.60%
Mother’s work		
Housewife	109	71.20%
Employee	43	28.80%
Number of family members		
mean (SD)	7.46 (3.45)	
Residence		
Urban	139	91.40%
Rural	13	8.60%
Number of children per family		
mean (SD)	4.33 (2.88)	
Home type		
Apartment	76	50%
Villa	64	42.10%
Popular	12	7.90%
Economic status		
Poor	15	9.90%
Good	119	78.30%
Excellent	18	11.80%
Father’s age (years)		
mean (SD)	39.16 (9.56)	
Mother’s age (years)		
mean (SD)	32.11 (7.55)	
Awareness of first aid measures		
Yes	7	4.60%
No	145	95.40%
Poisoning incidence		
Occurrence		
Yes	44/152	28.90%
No	108/152	71.10%
Children age (years) at poisoning incidence		
mean (SD)	3.29 (2.22)	
Mode of ingestion		
Self-ingestion	28/44	63.60%
Accidental ingestion	13/44	29.60%
Non-accidental ingestion	3/44	6.80%
Discovery		
Manifested	20/44	45.50%
Witnessed	5/22	22.70%
Informed	10/44	20.50%
Accidental	5/44	11.40%
Action taken		
Hospital transfer	36/44	81.80%
First aid measures	7/44	15.90%
No action	1/44	2.30%
Home medication history		
Presence		
Yes	130	85.50%
No	22	14.50%
Forms^**^		
Pills	82	53.90%
Syrups	68	44.70%
Capsules	2	1.30%
Storage place		
Refrigerator	65	42.50%
Pharmacy	64	41.80%
Other place	22	14.40%
Accessibility to kids		
Yes	76	50%
No	76	50%
Secure closure		
Yes	67	43.80%
No	85	55.60%
Possible risk factors		
Social problems		
Yes	24	15.70%
No	128	83.70%
Large number of family members^***^		
Yes	22	14.40%
No	130	85.52%
Children cared for by the in-house Nanny		
Yes	15	9.80%
No	137	89.50%
Is your child hyperactive?		
Yes	31	20.30%
No	121	79.10%

The study included 152 participants who responded to the questionnaire and provided their consent, 62 men (40.79%) and 90 women (59.21%). Among the respondents, the fathers’ mean (SD) age was 39.16 (9.56) years, while the mothers’ mean (SD) age was 32.11 (7.55) years. 28.90% reported the occurrence of poisoning among their children. Furthermore, self-ingestion was reported to be the most common mode of poisoning 28/44 (63.6%), followed by accidental ingestion 3/44 (29.6%) and non-accidental ingestion 3/44 (6.8%). Discovery of children poisoning was reported to be through the appearance of clinical manifestations suggesting poisoning 20/44 (45.5%), followed by witnessed ingestion 10/44 (22.7%), informed ingestion either by the child or another individual 9/44 (20.5%), and accidental discovery 5/44 (11.4%). Further, 36/44 (81.8%) of the respondents with a positive history of children's poisoning in their family transferred their poisoned child to a hospital; 7/44 (15.9%) performed first aid measures at home; and only 1/44 (2.3%) did not take any action.

Table 2 shows the comparison of the different studied variables in relation to the incidence of poisoning. Family history of childhood poisoning was positive in 44/152 (28.9%) of the respondents. Housewives (mothers) reported a significantly higher incidence of children poisoning than the employees: 33.9% vs. 16.3% respectively; P = .02. Moreover, respondents with a positive family history of children poisoning significantly reported a large number of family members, hyperactive children, and mouthing habits, as a claimed risk factors for poisoning (65.9%), (23.6%), and (65.9%) respectively; P was .000 for all). No significant differences were found regarding the other studied variables.

**Table 2:** Bivariate analysis of the respondents' characteristics in relation to incidence of poisoning * P-value significant <0.05; ** More than five family members

Studied variables	Incidence of poisoning				P-value^*^
	No (n = 108)		Yes ( n = 44)		
	Number	Percentage	Number	Percentage	
Mother’s educational status					
Educated	48	70.6%	20	29.4%	0.52
Not educated	60	71.4%	24	28.6%	
Mother’s work					
Housewife	72	66.1%	37	33.9%	0.022
Employee	36	83.7%	7	16.3%	
Housemaid:					
Yes	47	68.1%	22	31.9%	0.291
No	61	73.5%	22	26.5%	
Caregiver:					
Mother	78	74.3%	27	25.7%	0.132
Others	30	63.8%	17	36.2%	
Home area:					
Urban	98	70.5%	41	29.5%	0.449
Rural	10	76.9%	3	23.1%	
Having a family physician					
Yes	27	79.4%	7	20.6%	0.157
No	81	68.6%	37	31.4%	
Economic status					
Poor	11	73.3%	4	26.7%	0.111
Fair	88	73.9%	31	26.1%	
Excellent	9	50.0%	9	50.0%	
Marital status					
Married	96	71.6%	38	28.4%	0.833
Divorced	3	75.0%	1	25.0%	
Second wife	9	64.3%	5	35.7%	
Claimed risk factors for poisoning					
Large number of family members^**^					
Yes	6	5.5%	29	65.9%	0.000
No	102	94.5%	15	34.1%	
Hyperactive child					
Yes	14	12.9%	28	63.6%	0.000
No	94	87.1%	16	36.4%	
Mouthing					
Yes	10	9.3%	29	65.9%	0.000
No	98	90.7%	15	34.1%	

**Figure 1:**
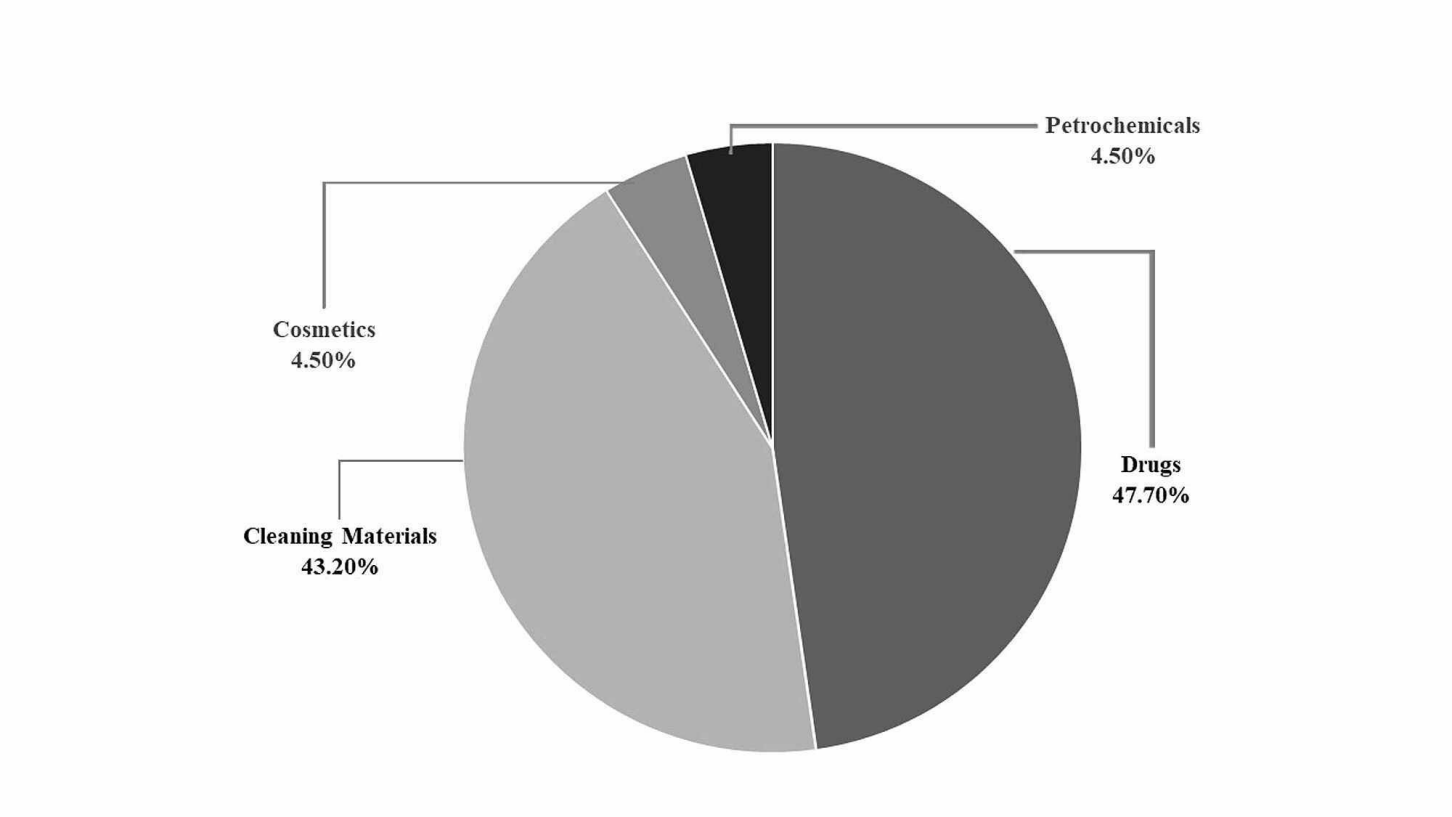
Causative materials for poisoning among the studied participants

Figure 1 shows the causative materials for poisoning among the studied participants. Medications were the most common causative agent reported for poisoning 21/44 (47.7%), followed by cleaning materials 19/44 (43.2%), and cosmetics 2/44 (4.5%), and lastly petrochemical materials 2/44 (4.5%).

## Discussion

Acute poisoning is one of the preventable causes of morbidity and mortality in children and still presents as a worldwide health problem considering the variability in the nature and type of poison consumed owing to variable accessibility and sociodemographic characteristics [12].

The current study showed that 44/152 (28.9%) of the respondents reported childhood poisoning in their family. This finding is comparable to those of another study in which admissions for accidental children home poisoning in a main hospital in Riyadh City accounted for 5.6% of the total annual admissions [8]. Moreover, Al Hazmi also reported an incidence of childhood drug intoxication of 7.4% in the pediatric medical ward and intensive care unit of a healthcare facility at Jeddah, Kingdom of Saudi Arabia during a period of two years (1994-1996) [13]. Furthermore, another study reported that the incidence of accidental childhood poisoning over a period of seven years (1992-1998) was 1.7% in Hafr Al Batin City, Kingdom of Saudi Arabia [14].

Our findings reflect the diversity of populations visiting King Khalid University Hospital and living in Riyadh City. However, such results cannot represent the exact incidence of childhood poisoning among the overall habitants of Riyadh City. Furthermore, there is no data registry for childhood poisoning in Riyadh City, which might highlight the need for regional or national programs for childhood poisoning.

The mean (SD) age of children with a history of poisoning in the current study was 3.29 (2.22) years. Conversely, Al Hazmi reported that the 2-5-year age group (61%) had a higher incidence of poisoning than the 0-2-year (27%), 5-10-year (9%), and 10-12-year age groups (3%) [13]. Moreover, another study from Saudi Arabia reported that 63% of the studied poisoned children were at the age of 1 to 3 years [14]. However, poisoning can occur in children younger than one year when the infants’ ability to explore is limited by their mobility or in children older than five years presumably because these children are becoming more discriminative and selective on what they swallow.

The current study found no sex preponderance for poisoning among the respondents who reported a positive family history of children's poisoning. Such findings are similar to those of another study that reported no sex difference regarding childhood poisoning [12]. However, some reports have documented male sex preponderance in childhood poisoning [15,16]. Moreover, a male-to-female sex ratio of 1.4:1 among children younger than 10 years and 3:1 among those aged 10-12 years was reported [13]. It is possible that under the age of five years, both boys and girls tend to have similar characteristics and behavioral patterns. Such variations might be explained by the differences in the sociodemographic characteristics of different communities.

Studies from different regions within the Kingdom of Saudi Arabia have reported variations in the causative agent for childhood poisoning. In the current study, medications were the most frequently reported causative agent for childhood poisoning among the studied respondents (48.8%), followed by cleaning materials (43.2%) and cosmetics and petrochemicals (each 4.5%). These findings are similar to those of other studies from different regions within the Kingdom of Saudi Arabia [8,11]. Similar studies from other countries have also reported drugs to be the most common causative agent [12,15]. Conversely, other studies have shown that hydrocarbon ingestion accounted for the highest proportion of poisonings (40%), followed by drug (34.3%) and household chemical ingestion (16%) [10,13].

Furthermore, significant variations in the type and nature of consumed poisons among developed and underdeveloped countries exist. Moreover, the availability and accessibility of potential poisonous materials, including pharmaceuticals and medication prescriptions, vary with variations in demography, education, awareness, social beliefs and habits, and socioeconomic status of families [12].

Such differences might be attributed to the variations in the commonly used household products, easy availability of medications over the counter in some countries, and differences in the awareness on the proper storage of such potentially risky materials for childhood poisoning among caregivers and families.

Another possible factor is that medications can be easily found inside homes in the Kingdom of Saudi Arabia because drugs can be purchased over the counter, and more medications might be prescribed with the possibility of multiple physician visits for the same illness. This highlights the importance of more regulations and restrictions of medications release by pharmacies, which may reduce children's easy access to medications at home.

In the current study, self-ingestion of poisonous materials was the most predominant method of poisoning, followed by accidental and non-accidental methods. Such findings are comparable to those of another study that reported childhood poisoning as accidental in the majority of their cases with occasional suicidal cases in adolescents [12]. Similarly, non-intentional poisoning was reported to be common among children [17].

The clinical manifestations and consequently the possible discovery of poisoning might depend on many factors, such as the type, nature, amount, and toxic effects of the causative agent. Another factor might be whether the event of ingestion of the child was witnessed or seen by another individual who might inform the caregivers. In the current study, the appearance of clinical manifestations suggesting poisoning was the most frequently reported method of poisoning discovery. These results are comparable to those of a previous study in which the clinical manifestations of poisoning were reported as a predominant key for poisoning recognition [12].

In the current study, the majority of respondents reported transferring their poisoned children to a hospital with the recognition of their poisoning. This finding is similar to those of another study [18]. Conversely, these findings are in contrast to those of another study that reported that only 20% of poisoned children were presented to a health care facility emergency within one hour [12]. The differences might be attributed to the geographic location of the patients and the availability of health care facilities in their areas. Another factor that might be considered is the poisoned child's clinical status and symptomatology of the poison, as the asymptomatic or mild cases might not be inciting factor pushing the caregiver to transfer the child seeking medical advice. More educational campaigns about childhood poisoning and management are warranted, as a research from Saudi Arabia showed that raising awareness among parents could reduce the risks posed to vulnerable children [19].

Limitations

Our study had a limited number of participants, which may not reflect the whole population living in different regions of Riyadh City. Hence, interpretation of the study results may not represent the different regions of the city. Another fact to observe is that the Arabic survey could have missed some non-Arabic participants presented to this hospital. A potential area for future research is to compare these results after the COVID-19 crisis with home-schooling and quarantine effect on poisoning among children.

## Conclusions

Our questionnaire survey revealed that accidental and non-intentional self-ingestion still presents as a major cause of childhood home poisoning. Despite the significant advancement in the life style among the majority of Saudi Arabian regions, especially the capital city, Riyadh, childhood poisoning is still a significant cause of morbidity and possible mortality. Creating health education and prevention programs might help prevent such a potentially serious preventable problem.

Nationwide studies are highly recommended to clarify more about the current incidence and potential risk factors for childhood poisoning. Furthermore, more utilization for the different channels for education on childhood safety might help reduce the incidence of such serious problems.
